# Maternal exposure to air pollution before and during pregnancy related to changes in newborn's cord blood lymphocyte subpopulations. The EDEN study cohort

**DOI:** 10.1186/1471-2393-11-87

**Published:** 2011-11-02

**Authors:** Nour Baïz, Rémy Slama, Marie-Christine Béné, Marie-Aline Charles, Marie-Nathalie Kolopp-Sarda, Antoine Magnan, Olivier Thiebaugeorges, Gilbert Faure, Isabella Annesi-Maesano

**Affiliations:** 1 Inserm, Institut national de la santé et de la recherche médicale, Epidemiology of Allergic and Respiratory (EPAR) Department, UMR-S707, Paris, France; 2 Faculté de Médecine de Saint-Antoine, UPMC Univ6, Paris, France; 3 Inserm, U823, Team "Environmental Epidemiology applied to Reproduction and Respiratory Health", Institut Albert Bonniot, Grenoble, France; 4 Université J. Fourier Grenoble, Grenoble, France; 5 Laboratoire d'Immunologie, CHU de Brabois & Nancy Université, Nancy, France; 6 Institut national de la santé et de la recherche médicale, UMR 780, Villejuif, France; 7 Institut national de la santé et de la recherché médicale, U684, Vandoeuvre les Nancy, France; 8 Institut national de la santé et de la recherche médicale, UMR 915, Institut du Thorax, IRT-UN, Nantes, France; 9 Service de Gynécologie-Obstétrique, Maternité de Nancy, Nancy, France

## Abstract

**Background:**

Toxicants can cross the placenta and expose the developing fetus to chemical contamination leading to possible adverse health effects, by potentially inducing alterations in immune competence. Our aim was to investigate the impacts of maternal exposure to air pollution before and during pregnancy on newborn's immune system.

**Methods:**

Exposure to background particulate matter less than 10 μm in diameter (PM_10_) and nitrogen dioxide (NO_2_) was assessed in 370 women three months before and during pregnancy using monitoring stations. Personal exposure to four volatile organic compounds (VOCs) was measured in a subsample of 56 non-smoking women with a diffusive air sampler during the second trimester of pregnancy. Cord blood was analyzed at birth by multi-parameter flow cytometry to determine lymphocyte subsets.

**Results:**

Among other immunophenotypic changes in cord blood, decreases in the CD4+CD25+ T-cell percentage of 0.82% (p = 0.01), 0.71% (p = 0.04), 0.88% (p = 0.02), and 0.59% (p = 0.04) for a 10 μg/m^3 ^increase in PM_10 _levels three months before and during the first, second and third trimester of pregnancy, respectively, were observed after adjusting for confounders. A similar decrease in CD4+CD25+ T-cell percentage was observed in association with personal exposure to benzene. A similar trend was observed between NO_2 _exposure and CD4+CD25+ T-cell percentage; however the association was stronger between NO_2 _exposure and an increased percentage of CD8+ T-cells.

**Conclusions:**

These data suggest that maternal exposure to air pollution before and during pregnancy may alter the immune competence in offspring thus increasing the child's risk of developing health conditions later in life, including asthma and allergies.

## Background

Evidence is accumulating of close connections between *in utero *exposure to toxicants and the development of the immune system, the impairment of which is involved in the development of various health conditions [1]. In recent years, there have been a growing number of arguments showing that susceptibility to disease is largely set *in utero *or early in infancy [2]. Asthma [3], elevated immunoglobulin E (IgE) [4] and mortality in children [5] and adults [4,6] may be determined *in utero *and early life. Several population-based studies have associated exposure to urban air pollution during pregnancy with birth outcomes, including impaired fetal growth [7], low birth weight and premature birth [8-10], postneonatal infant mortality, intrauterine growth restriction and a decreased lung function [11] as well as allergies [12] and asthma [13] in childhood. A link has also been shown between maternal exposure to environmental tobacco smoke and a low birth weight [14], decreased lung function as well as allergies and asthma in childhood [15,16].

Furthermore, thymus functions are altered by exposure during pregnancy to environmental immunotoxicants, such as 2, 3, 7, 8-tetrachlorodibenzop-dioxin (TCDD) and polychlorobiphenyls (PCB) in animals [17] and humans [18]. Few studies have related maternal exposure to toxicants during pregnancy and cord blood cells involved in organism defenses. Significant changes in immunological variables in cord blood of babies from smoking mothers compared to those of non-smoking mothers have been shown [15,19]. Similarly, living in a highly polluted urban area was related to a lower percentage of CD4+ T-lymphocytes and a lower CD4+/CD8+ ratio but a higher percentage of NK cells [20] in the cord blood among newborns from the Czech Republic. More recently, maternal exposure to polycyclic aromatic hydrocarbons (PAHs) and fine particulate matter less than 2.5 μm in diameter (PM_2.5_) assessed during the 14 days before birth in two districts in the Czech Republic was associated with lower percentages of CD3+, CD4+ and CD8+ T-lymphocytes and a higher percentage CD19+ B-lymphocytes in newborns [21]. However, such investigations have been limited by ecological approaches, exposure misclassification, limited sample size and failure to take potential confounders of the relationship into account.

The general objective of our work was to investigate whether maternal exposure to air pollution before and during pregnancy intervenes in the development of the newborn's immune cells. Our study was conducted in the frame of the French EDEN mother-child cohort.

## Methods

### General study scheme, study population and exclusion criteria

Mother-children pairs were recruited in the EDEN (Pre and postnatal determinants of the child's development and health) prospective Birth Cohort Study http://ifr69.vjf.inserm.fr/~webifr/etude_EDEN.html. The primary aim of the EDEN Cohort is to identify prenatal and early postnatal nutritional, environmental and social determinants associated with children's health and their normal and pathological development. Pregnant women seen for a prenatal visit at the departments of Obstetrics and Gynecology of the University Hospital of Nancy and Poitiers before their twenty-fourth week of amenorrhea were invited to participate. Enrolment started in February 2003 in Poitiers and September 2003 in Nancy; it lasted 27 months in each centre and allowed the inclusion of 2002 women. Women with speaking and writing abilities in French, who did not suffer from insulin dependent diabetes, did not plan to deliver outside the university hospital or move out of the region within the 3 years and who benefited of the social security system, were included in the study. Multiple pregnancies were excluded. Our investigation was conducted in 370 mothers-newborns pairs, who were recruited from the city of Nancy and for whom newborns' cord blood was available. Women whose delivery occurred on weekends were excluded from our study population, since blood samples were analyzed on the day of delivery. Information about blood collection and analysis will be provided below.

Women were given an appointment with a study midwife, planned to take place between 24 and 28 gestational weeks, during which an interview on behavioral factors was conducted and biological samples were collected. Further information on the mothers and their newborns, including perinatal infections, mode of delivery, newborn's sex and weight, gestational age and season of birth, were either collected by a questionnaire after birth or extracted from clinical record.

### Lymphocyte immunophenotyping

We measured CD19+ B-cells, CD16+56+ natural killer (NK) cells, CD3+ T-cells, CD4+ T- cells, CD8+ T-cell, and CD4+CD25+ T-cells to characterize the degree of impairment of the innate and acquired immune system. Based on evidence from previous studies showing that T-cells [22] as well as NK cells [23] play a critical role in the pathogenesis of allergy and asthma, we chose to focus on these lymphocyte subpopulations. In order to assess the relative proportion of each lymphocyte subset, we performed lymphocyte immunophenotyping at delivery according to standardized procedures [24]. In order to prevent any contamination with maternal blood, the cord was doubly clamped immediately after birth (vaginal delivery) or after extraction of the fetus through the uterine incision (elective caesarean section), repeatedly rinsed and venous cord blood was sampled between the two clamps. Problems with contamination of cord blood lymphocytes with nucleated red blood cells were avoided by a lysed whole blood method [25]. Briefly, we incubated whole fresh blood aliquots of 50 μL, conditioned into heparinized Vacutainers, with antibody mixtures (reagents from Beckman Coulter, Miami, FL) for 15 minutes at +4°C in the dark. Red blood cells were then lysed with Immunoprep (Beckan Coulter) and fixed in paraformaldehyde. Blood samples were stored at +4°C in polystyrene boxes and transported in coolers for analysis at the Clinical Immunology Division, Hospital of Nancy (Pr Gilbert Faure). We processed the samples with flow cytometry using an EPICS-XL4 (Beckman Coulter). Applying gating strategies using the whole lymphocytic population identified on a FSC/SSC size/structure scatter-gram made it possible to express the frequencies of each subset as a percentage of total lymphocytes. All samples were analyzed on the day of delivery, upon arrival at the laboratory, with no difference in time until processing, and standard operating procedures for each assay were established.

### Maternal exposure to air pollutants

We assessed maternal exposure to background dioxide nitrogen (NO_2_) and particulate matter less than 10 μm in diameter (PM_10_) by attributing representative concentrations provided by permanent stations closest to the mothers' residence address after geolocalisation through Geographic Information System (GIS) [26]. We calculated mean concentrations of the pollutants three months before the beginning of pregnancy and for each trimester of pregnancy and used them in the analysis. In a sub-sample of 60 non-smoking mothers, we assessed personal exposure to four volatile organic compounds, benzene, toluene, ethylbenzene and xylenes (BETX), with a diffusive air sampler (Radiello, Fondazione Salvatore Maugeri-Centro di Ricerche Ambientali, Padova, Italy) which relies on radial symmetry diffusion [27]. The cylindrical diffusive body contains a stainless steel net cylindrical cartridge, filled with activated charcoal. Passive samplers were carried for seven consecutive days during the second trimester of pregnancy [7]. Women were asked not to touch the diffusive air sampler with their hands, avoid contact with water, always carry the air sampler with them, attaching it on their clothes as closely as possible to their collar, and to keep it close to their bed when they slept. We excluded women for whom the diffusive part of the sampler was broken during use (n = 4). The women stored the absorbing cartridges in a capped glass tube before and after the 7-day exposure period they sent them to Institut National de la Santé et de la Recherche Médicale (INSERM) by post after use along with a questionnaire on the conditions of use. We temporarily stored the charcoal cartridges and we shipped them to the Maugeri Foundation, where they were stored at 4°C before analysis. We shipped the cartridges with a bar code identifier and information on the hours and days of start and end of exposure to ambient air, but no information on pregnancy outcome. Collected vapors were desorbed from the cartridge using carbon disulfide solvent with a benzene concentration < 0.1 μg/mL and the solution analyzed using high-resolution gas chromatography with a flame ionization detector. Taking into account the actual number of hours of exposure of the dosimeter made it possible to convert the benzene concentration in the solution to a mean concentration in the air during the period of exposure. All these women were non-smokers, which made it possible to uniquely assess concentrations of BETX contained in outdoor and indoor air, including environmental tobacco smoke. The detection limit for an exposure of 5 days was 0.1 μg/m^3 ^for benzene.

### Statistical methods

We calculated frequencies of categorical variables and means and standard deviations of continuous variables. We fitted multivariate-adjusted linear models to examine the association between each air pollutant and each lymphocyte phenotype after controlling for potential confounders. Regression coefficients and confidence intervals provided by the multivariate-adjusted linear regression models made it possible to estimate the changes in lymphocyte percentages for a 10 μg/m^3 ^increase in PM_10 _and NO_2_, respectively, at different time-windows of exposure, and for a 1 μg/m^3 ^increase in log-transformed benzene exposure. In addition, with the purpose of avoiding multiple testing problems, we used a second-order linear orthogonal polynomial regression and assessed the relationship between the distributions of lymphocyte immunophenotypes on one hand and exposure to PM_10 _and NO_2 _on the other hand during the whole period of gestation. This polynomial regression allowed modeling the relationship by taking into account simultaneously the average value, the trend and the differences of health and exposure variables respectively at each trimester of pregnancy. To test linearity of the lymphocyte phenotypes, we performed linear trend tests with categorical (tertile) exposure variables. We retained the normality hypothesis for CD4+/CD8+ ratios and the distributions of all lymphocyte percentages except for NK cells that were log-transformed. NO_2_, PM_10 _and benzene were considered as continuous variables. Because of the skewed distribution of its exposure, benzene values were log-transformed. The Pearson correlation coefficient was calculated between NO_2 _and PM_10 _in the sample of 370 women, and between NO_2_, PM_10 _and benzene in the subgroup of 56 non-smoking women. Adjustment factors were chosen on the basis of their known relationship to immune system parameters, independently of any association with exposures. They included: mother's age at delivery and body mass index, maternal history of allergy, active and passive smoking, perinatal infections, mode of delivery including delivery via caesarean, prolonged labor, season of the birth, newborn's sex, weight and gestational age at birth, which are delivery factors that could influence lymphocyte immunophenotype distributions. Maternal allergic history was obtained regarding physician-diagnosed allergic diseases such asthma, rhinitis, eczema and food allergies. We performed all data analyses using SAS statistical software, version 9.1. P-values < 0.05 were considered statistically significant for all analyses.

### Ethics Statement

The study was approved by the relevant ethical committees (Comité Consultatif pour la Protection des Personnes dans la Recherche Biomédicale, Le Kremlin-Bicêtre University hospital, and Commission Nationale de l'Informatique et des Libertés), and all participating women gave informed written consent for themselves and for their child to be part of the study.

## Results

### Characteristics of the study population

Table 1 presents the characteristics of the sample of 370 women living within less than 2 km from a monitoring station, the subset of 56 women with BETX personal exposure assessments and their newborns and the full cohort (n = 2002) from which our study sample was selected. Compared to the full cohort, the total sample (n = 370) consisted of proportionally less mothers smoking during the first trimester of gestation, more winter and spring births and less preterm (birth at < 37 weeks completed weeks of gestation) and low birth weight (< 2, 500 grams) infants. The sample did not differ from the full cohort with respect to some maternal characteristics including age, body mass index, employment status, exposure to environmental tobacco smoke during pregnancy, active smoking before gestation, during the second and third trimester of pregnancy, maternal history of allergy and mean gestational duration, and newborn's sex. The comparison between the total study sample (n = 370) and the subgroup of 56 non-smoking women did not show any significant difference with respect to age, body mass index, employment status, percentage of women exposed to passive smoking during pregnancy, maternal history of allergy, gestational duration, birth weight, newborn's sex and season of birth. The only significant difference was observed in maternal active smoking during the first, second and third trimester of pregnancy (respective chi-square test P-value: 0.037; 0.001; 0.002). Indeed, the 56 women of the subgroup were selected according to their non-smoking status during pregnancy. In our study population, no mothers reported gestational diabetes or chronic illnesses apart from asthma, eczema and other allergic diseases, such as rhinitis and food allergies. One third of the 370 women were smoking before the beginning of their pregnancy. The proportion of smokers decreased in the first and second trimester of gestation and increased slightly in the third trimester (Table 1). Nearly a third of pregnant women were exposed to passive smoking during the two first trimesters and a quarter during the third trimester. Table 2 shows the distribution of lymphocyte subsets in the whole cohort and in the subsample of non-smoking mothers with BETX assessments. No significant differences were found between the two groups with respect to the proportions of the various lymphocyte subsets or to the CD4+/CD8+ ratio. When calculated in the study sample of 370 women, NO_2 _and PM_10 _measured three months before the beginning of pregnancy and for each trimester of gestation were positively correlated, thereby indicating a linear association between these pollutants (Table 3). However, in the subgroup of 56 non-smoking women, no significant correlation was found between NO_2_, PM_10 _measured for the second trimester and the volatile organic compounds (BTEX), except between each VOC (Table 4).

**Table 1:** Comparison of characteristics of mother-newborn pairs in full cohort versus subset with lymphocyte immunophenotype versus subgroup with Volatile Organic Compounds personal assessment and lymphocyte immunophenotype

Factor	Full cohort (*n *= 2002)	Total study sample (*n *= 370)	P-value (full cohort vs. total study sample)	Non-smoking mothers with BETX assessment (*n *= 56)	P-value (Total study sample vs. subset of 56)
**Mother's age**, mean ± SD, years	29.99 ± 4.89	29.77 ± 4.72	0.39	29.25 ± 3.64	0.37
19 - 24.9 (%)	11.71	9.88	0.26	3.7	0.12
25 - 34.9 (%)	70.95	74.55		87.04	
> 35 (%)	17.34	15.57		9.26	
**BMI**, mean ± SD, kg/m^2^	26.32 ± 4.46	25.89 ± 4.10	0.1	25.00 ± 3.37	0.13
Normal (%)	45.09	48.77	0.22	57.14	0.56
Overweight (%)	37.82	36.78		35.71	
Moderate obesity (%)	12.13	11.44		5.36	
Severe obesity (%)	3.69	1.91		1.79	
Morbid obesity (%)	1.27	1.09		0	
**Employment status**, %	71.23	74.12	0.17	78.57	0.48
Full-time	77.4	77.74	0.9	80	0.73
Part-time	22.6	22.26		20	
**Passive smoking**, %					
During 1^st ^trimester	30.68	32.25	0.47	28.57	0.58
During 2^nd ^trimester	28.62	28.73	0.96	25	0.56
During 3^rd ^trimester	26.21	25.75	0.82	25	0.91
**Active smoking **, %					
Before pregnancy	36.28	32.97	0.1	23.21	0.14
During 1^st ^trimester	25.84	20.71	0.01	0	0.04
During 2^nd ^trimester	17.55	15.26	0.19	0	0.001
During 3^rd ^trimester	16.73	17.12	0.82	0	0.002
**Maternal history of allergies**, %	51.74	51.1	0.78	51.79	0.92
**Gestational duration**, mean ± SD, weeks	39.22 ± 1.75	39.41 ± 1.22	0.54	39.45 ± 1.16	0.72
< 37 weeks (%)	6.17	2.43	0.004	1.79	0. 09
≥ 37 weeks (%)	93.83	97.57		98.21	
**Birth weight**, mean ± SD, g	3279 ± 585	3343 ± 456	0.05	3359 ± 396	0.58
< 2, 500 g (%)	10.61	2.43	< 0.0001	1.79	0.09
≥ 2, 500 g (%)	89.39	97.57		98.21	
**Newborn's sex **, %					
Male	52.55	49.19	0.15	57.14	0.27
Female	47.45	50.81		42.86	
Season of birth*, %					
Autumn	22.22	16.8	0.003	14.29	0.43
Winter	21.03	22.76		30.36	
Spring	30.17	36.59		39.29	
Summer	26.58	23.85		16.07	

SD, standard deviation; BTEX = benzene, ethylbenzene, toluene, xylenes; * calendar defined.

**Table 2:** Distribution of lymphocyte subsets

Lymphocyte immunophenotypes	Total study sample (*n *= 370) Mean* ± SD	Non-smoking mothers with BETX assessments (*n *= 56) Mean* ± SD
**CD3+ ***(%)*	58.35 ± 11.9	58.41 ± 15.04
**CD4+ ***(%)*	43.14 ± 10.30	43.46 ± 12.91
**CD8+ ***(%)*	15.98 ± 6.58	13.86 ± 5.26
**CD4+/CD8+**	3.19 ± 1.59	3.47 ± 1.54
**NK ***(%)*	10.46 ± 9.64	9.82 ± 12.49
**CD4+ CD25+ ***(%)*	7.41 ± 5.14	7.63 ± 2.33
**CD19+ ***(%)*	8.81 ± 5.11	**/**

* Mean % of sum of (CD3+, CD19+ and NK). SD, standard deviation; BTEX = benzene, ethylbenzene, toluene, xylenes. (/= missing data). Chi-square test p < 0.05.

**Table 3:** Spearman correlations for pollutants in the total study sample of 370 women

	NO_2_, bf	PM_10_, bf	NO_2_, t1	PM_10_, t1	NO_2_, t2	PM_10_, t2	NO_2_, t3	PM_10_, t3
**NO_2_, bf**	1.00	0.50	0.88	0.50	0.76	0.39	0.78	0.38
**PM_10_, bf**		1.00	0.44	0.72	0.51	0.67	0.46	0.46
**NO_2_, t1**			1.00	0.49	0.84	0.36	0.70	0.40
**PM_10_, t1**				1.00	0.48	0.65	0.49	0.61
**NO_2_, t2**					1.00	0.43	0.84	0.38
**PM_10_, t2**						1.00	0.27	0.50
**NO_2_, t3**							1.00	0.49
**PM_10_, t3**								1.00

All correlations are significant at p < 0.0001; bf = three months before the beginning of pregnancy; t1 = 1^st ^trimester; t2 = 2^nd ^trimester; t3 = 3^rd ^trimester.

**Table 4:** Spearman correlations for pollutants in the subset of 56 non-smoking women with BTEX personal exposure assessment

	Benzene	Ethylbenzene	Toluene	Xylenes	PM_10_, t2	NO_2_, t2
**Benzene **	1.00	0.72 p < 0.0001	0.38 p = 0.0041	0.61 p < 0.0001	-0.017 p = 0.9118	-0.032 p = 0.8246
**Ethylbenzene**		1.00	0.30 p = 0.0240	0.95 p < 0.0001	-0.074 p = 0.6278	0.12 p = 0.4158
**Toluene**			1.00	0.23 p = 0.0827	0.073 p = 0.6346	-0.22 p = 0.1304
**Xylenes**				1.00	-0.078 p = 0.6078	0.17 p = 0.2490
**PM_10_, t2**					1.00	-0.003 p = 0.9835
**NO_2_, t2**						1.00

t2 = 2^nd ^trimester; BTEX = benzene, ethylbenzene, toluene, xylenes

### Maternal exposure to NO_2 _and PM_10 _and lymphocyte changes

**Figure 1:**
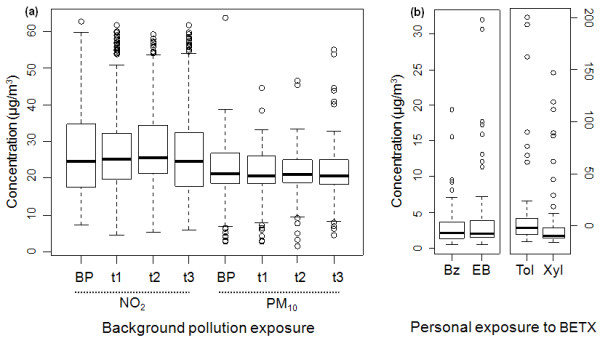
**Maternal exposure levels to air pollution**. (a) Box-plot of maternal exposure levels to background pollutants three months before pregnancy (BP) and during each trimester of pregnancy (t1, t2, t3) in the total sample of 370 mothers. (b) Box-plot of maternal exposure to benzene, ethylbenzene, toluene and xylenes for seven consecutive days of the second trimester in the sub-group of 56 non-smoking mothers with BETX (Benzene, Ethylbenzene, Toluene, Xylenes) assessment. The bottom and top edges of the boxes are located at the first quartile and third quartile of the sample. Within the boxes, the median is displayed as a line (second quartile). The circles displayed beyond upper and lower fences are outliers and stand for values three times above or below the interquartile range (the difference between the first and third quartile). Bz = benzene; EB = ethylbenzene; Tol = toluene; Xyl = xylenes.

Average maternal exposure to background air pollutants was stable from one trimester to another (Table 5). Figure 1a shows the distribution of maternal exposure levels to background pollutants three months before pregnancy and during each trimester of pregnancy in the total sample of 370 mothers. In addition, some mothers were exposed to very high levels of the assessed BETX (Figure 1b). Adjusted linear regression models showed that CD8+ T-cell and NK cell percentages increased significantly with PM_10 _(respectively 3 months before pregnancy and during the first trimester) and NO_2 _(respectively during the two first trimesters and during the third trimester) concentrations (Table 6). Conversely, CD3+ and CD4+CD25+ cells and the CD4+/CD8+ ratio decreased as PM_10 _exposure increased (Table 6). Results were confirmed when applying the orthogonal polynomial regression model for 3 values. In particular, the negative association between PM_10 _exposure and CD4+CD25+ T-cells strongly persisted (p = 0.0086). The results persisted after adjustment for the confounders, including perinatal infection and season of birth. No significant association was found between exposure to air pollutants and the percentages of CD19+ B-lymphocytes and CD4+ T-cells (data not shown).

**Table 5:** Maternal exposure to background pollutants and BETX

	Total study sample		Non-smoking mothers with BETX assessment			
**Mean ± SD (μg/m^3^)**	NO_2_	PM_10_	Benzene	Toluene	Ethylbenzene	Xylenes*
	
**Window of exposure time**	n = 370		n = 56			
	
*Before pregnancy*						
3 months bf	27.6 ± 13.0	22.0 ± 6.7				
*During pregnancy*						
1^st ^trimester	28.2 ± 12.7	21.8 ± 5.8				
2^nd ^trimester	28.2 ± 11.3	21.8 ± 5.2	3.3 ± 2.0	41.5 ± 16.5	5.1 ± 2.0	23.3 ± 8.7
3^rd ^trimester	26.7 ± 12.1	21.9 ± 6.4				

* Sum of o-xylene, m-xylene and p-xylene. SD, standard deviation; bf, before; BETX, benzene, ethylbenzene, toluene, xylenes.

**Table 6:** Relationships between lymphocyte subset percentages in cord blood of newborns and air pollution exposure according to the window of exposure

	CD3+	CD8+	CD4+/CD8+	CD4+CD25+	CD19+	Log(NK)
***n *= 370**	*β (95% CI) *^§^	*β (95% CI)*	*β (95% CI)*	*β (95% CI)*	*β (95% CI)*	*β (95%CI)*

Air pollutant* **and window of exposure**						
***3 months before pregnancy***						
NO_2_	-0.27 (-1.45; 0.90)	0.47 (-0.20; 1.13)	-0.07 (-0.23; 0.09)	0.03 (-0.30; 0.24)	2.50 (-0.37; 0.87)	0.05 (-0.03; 0.12)
PM_10_	-1.32 (-3.97; 1.33)	1.52 (0.05; 2.99) ^‡^	-0.23 (-0.57; 0.04) ^†^	-0.82 (-1.45; -0.19) ^‡^	-0.20 (-1.88; 1.51)	0.14 (-0.05; 0.55)
***During pregnancy***						
NO_2 _in 1^st ^trimester	0.19 (-0.96; 1.34)	0.63 (-0.01; 1.27) **^†^**	-0.07 (-0.22; 0.08)	-0.14 (-0.40; 0.12)	-0.09 (-0.66; 0.48)	0.03 (-0.05; 0.13)
NO_2 _in 2^nd ^trimester	0.49 (-0.84; 1.82)	0.68 (-0.02; 1.44) **^†^**	-0.002 (-0.17; 0.17)	-0.08 (-0.38; 0.22)	-0.27 (-0.92; 0.38)	0.02 (-0.07; 0.12)
NO_2 _in 3^rd ^trimester	0.03 (-1.30; 1.35)	0.55 (-0.20; 1.30)	-0.02 (-0.19; 0.15)	-0.17 (-0.47; 0.12)	0.11 (-0.51; 0.73)	0.09 (0.01; 0.17) ^‡^
PM_10 _in 1^st ^trimester	-3.10 (-6.02; -0.18) ^‡^	0.85 (-0.77; 2.49)	-0.17 (-0.55; 0.21)	-0.71 (-1.37; -0.05) ^‡^	0.33 (-1.35; 2.01)	0.22 (0.008; 0.43) ^‡^
PM_10 _in 2^nd ^trimester	0.31 (-2.96; 3.57)	0.22 (-1.53; 1.97)	-0.05 (-0.55; 0.20)	-0.88 (-1.60; -0.17) ^‡^	-0.63 (-2.29; 1.02)	-0.10 (-0.33; 0.13)
PM_10 _in 3^rd ^trimester	0.56 (-1.90; 3.01)	1.09 (-0.26; 2.44)	-0.06 (-0.26; 0.31)	-0.59 (-1.15; -0.04) ^‡^	0.35 (-0.84; 1.53)	0.05 (-0.12; 0.24)

* Continuous variables.

^† ^0.05 < p < 0.09.

^‡ ^p < 0.05.

^§ ^β (95% CI): adjusted regression coefficient and 95% confidence interval for mother's age and body mass index, maternal history of allergy, active and passive smoking (data questionnaires), perinatal infections, mode of delivery, newborn's sex and weight, gestational age at birth and season of birth (data from postnatal questionnaire). β corresponds to the change in lymphocyte percentages (log-transformed for natural killer cells) for each 10 μg/m^3 ^increase in PM_10 _or NO_2_.

### Maternal exposure to benzene and lymphocyte changes

**Figure 2:**
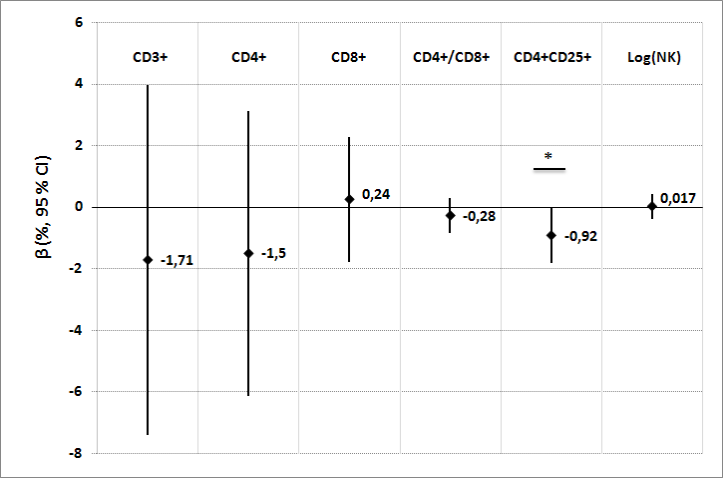
**Associations between lymphocytes percentages and benzene exposure**. Relationships between lymphocyte subset percentages in cord blood of newborns and benzene exposure in the sub-group of 56 non-smoking mother-newborn pairs with BETX (Benzene, Ethylbenzene, Toluene, Xylenes) assessment. β (95% CI): adjusted regression coefficient and 95% confidence interval for mother's age and body mass index, maternal history of allergy, passive smoking, perinatal infections, mode of delivery, newborn's sex and weight, preterm birth and season of birth). β corresponds to the change in lymphocyte percentages (log-transformed for natural killer cells) for each increase in one in log-transformed benzene exposure (n = 56). * p = 0.05.

After adjustment for confounding factors (Figure 2), an increase in 1 in log-transformed benzene exposure was associated with a significant decrease in CD4+CD25+ T-cells. No significant association was found between toluene, ethylbenzene or xylenes and lymphocyte immunophenotypes (data not shown).

## Discussion

This study indicates that ambient air pollution to which the mother is exposed before and during pregnancy might alter the relative distribution of cord blood lymphocyte phenotypes in her newborn, thus possibly influencing the future child's health. Fetal blood CD8+ and NK cells increased whereas CD3+, CD4+CD25+ and CD4+/CD8+ ratio decreased as maternal exposure to PM_10 _and NO_2 _the three-month period before and during pregnancy increased. Similarly, CD4+CD25+ T-cells decreased while maternal exposure to benzene during the second trimester of pregnancy increased, as assessed using a personal diffusive air sampler.

Our findings extend previous data from the rare studies having investigated the links between prenatal exposure to air pollution and the immune system. In the first study carried out in the Czech Republic, living in polluted areas was found to be associated with a decrease in CD3+ and CD4+ T-cells and in the CD4+/CD8+ ratio, as well as with a higher proportion of NK cells in fetal blood [20]. Our study also found an inverse relationship between PM_10 _exposure and the CD4+/CD8+ ratio, although only at the borderline level (p = 0.08). Successively, the same group showed short-term associations between PAHs and PM_2.5 _and immune parameters in Czech newborns [21]. Average PAH or PM_2.5 _levels during the 14 days before birth were associated with significant decrements in the T-lymphocyte phenotype fractions (i.e. CD3+, CD4+ and CD8+) and a clear increase in the B-lymphocyte (CD19+) fraction in cord blood. In spite of the similarities in the results, Hertz-Picciotto *et al*. studies [20] and ours differ in the concentrations to which the mothers were exposed that were very high in the Czech area and comparable to current World Health Organization standards in our study and in the estimation of maternal exposure that was both geo-localized and assessed with personal samplers in our investigation. More recently, in agreement with our observations, children exposed to elevated air pollution in Mexico City exhibited significantly increased numbers of CD8+ cells (p = 0.02) [28]. However, these children also exhibited significant decreases in the numbers of NK cells (p = 0.003), which contrasts with our results. However, in the Mexican investigation the children were seen at 10 years of age and no cord blood was available. The Mexican study thus targeted a window of exposure that was different from the one considered in our study. In addition, a recent study carried out in children showed that increased exposure to ambient air pollution was associated with the impairment of Treg cells function and may increase asthma morbidity [29].

No previous study has related regulatory T-cells in early life to exposure to air pollution in spite of the fact that they have been involved in the development of allergies [30]. We targeted CD4+CD25+ T-cells as physiologically generated CD4+CD25+ T-cells, which are the most widely studied type of regulatory T-cells (Tregs), especially in newborns where most cord blood CD4+CD25+ T-cells correspond to regulatory T-cells [31]. In our birth cohort study, decreased CD4+CD25+ T-cell levels were significantly correlated to PM_10_, benzene and moderately to NO_2 _indicating that by decreasing Treg cells, pollutant exposure might increase the risk of allergy in newborns. Indeed, regulatory T-cells can inhibit the development of allergic Th2 responses and could thus play an important role in the development of allergy [32]. As further confirmation, it has recently been shown that Treg cells are defective in allergic subjects [32,33] including asthmatics [30,34,35], and impaired regulatory T-cells were found in cord blood of newborns at hereditary risk of allergy [35,36]. This situation is consistent with the involvement of air pollution in the so-called "allergy epidemics" observed in past decades as traffic-related pollution increased. The fact that the relationship was stronger for particulate matter further supports this hypothesis on the basis of experimental evidence having directly related particulate matter to the development of allergy [37]. The observed CD4+CD25+ T-cells deficiency could result from the effect of exposure to air pollution on the Treg compartment or as a consequence of pro-inflammatory mechanisms driven by pollutant exposure and leading to an increase in CD8+ T-cells and NK cells. Latent viral infections could also be involved in this process [38].

Besides, some studies have suggested the probable contribution to asthma pathogenesis of CD8+ T-cells producing IL4 and IL5 (type 2 CD8+ T-cells) in the lungs and blood of asthmatic individuals [39]. CD8+ T-cells producing IFN-γ (type 1 CD8+ T-cells) may also contribute to asthma symptoms and to asthma exacerbation in individuals suffering already from asthma [40], and this in spite of the fact that in early life they may protect against asthma development, by eliminating allergen-specific Th2 cells. In addition, virus-specific CD8+ T-cells producing type 2 cytokines may develop during certain viral infections, suggesting a mechanism for virus-induced asthma exacerbation [41,42]. Recently, Natural Killer T (NKT) cells comprising a subpopulation of lymphocytes that express features of NK cells and conventional T-cells have been suggested to play an important pathogenic role in asthma [43]. A role for subsets of NKT cells in asthma has been suggested by extensive studies in animal models of asthma induced with allergen, viral infection, ozone exposure, or bacterial components, suggesting that NKT cells function in causing airway hyperreactivity. The clinical relevance of NKT cells in human asthma is supported by the observation that NKT cells are present in the lungs of some patients with asthma, particularly patients with severe, poorly controlled asthma. However, the validation of the concept that NKT cells may contribute to asthma still requires additional human studies. Thereafter our findings in a sample of mother-child pairs are consistent with previous experimental studies.

Regarding maternal exposure to organic volatile compounds (VOCs) that was objectively assessed in our study, our results are consistent with the findings of one study that showed associations between maternal exposure to VOCs and an altered cytokine secretion profile of cord blood T-cells [44]. In particular, the authors found a diminished capacity of cord blood T-cells to produce the Th1 cytokine IFNγ in infants from exposed mothers, which is a risk factor for the development of allergic disease in infancy [45,46].

An originality of our work was the systematic assessment, along with the whole CD4+ compartment, of CD4+CD25+ T-cells that have been described as a "proxy" of regulatory T-cells (Tregs) [47]. However, we have to be cautious in interpreting our findings because we did not have at the time of the survey at our disposal the assessment of forkhead/winged-helix transcription factor FoxP3 [48,49] which would have been more accurate in order to identify regulatory T-cells. In addition, we did not estimate functional immune parameters that were deemed not feasible in our project because the quality of these measurements degrades with storage time and transportation. Another strength of our study is that we geo-localized the child's address, using Geographic Information System (GIS) technology, in order to attribute to each child representative values of background air pollution [7] and thus reduce exposure misclassification [50]. In addition, for the assessments of exposure to benzene, the mothers carried personal samplers, that allow taking into account both indoor and outdoor exposures to benzene including microenvironments such as vehicle cabins [51]. An additional gist of our investigation consists in the robustness of the results as children were drawn from the general population and their parents could provide information on possible confounders, thus diminishing the probability of having obtained random results. However, considering background air pollution assessment, although performed by monitoring stations located in close proximity of the mothers' residence, could have led to underestimation of exposure in our investigation. In addition, some cells were privileged compared to others, which opens the way to other studies. Although the distributions of some variables were significantly different in the study sample (n = 370) compared with the full cohort (e.g., the proportion of low birth weight newborns was 2.43% in the study total sample and 10.61% in the full cohort), these differences are not likely to have affected either the internal or external validity of the results, because the variables were controlled in the analysis through adjustment in the multivariate models. Lastly, as an additional positive element, we avoided the multiple testing problem by using a second-order linear orthogonal polynomial regression that yielded similar results, strongly significant in the case of PM_10 _exposure and CD4+CD25+ T-cells. This had never been done previously for the period of pregnancy.

The findings on the effects of maternal exposure to air pollution during pregnancy on lymphocyte immunophenotype distributions in cord blood, even at low air pollutants levels, suggest that the fetus might be highly sensitive to maternal exposures and its variations during pregnancy. Moreover, it is frequently surmised that prenatal and early life exposures to air pollution are of greater significance than later exposure due to the susceptibility of target organs and systems during developmental periods of life [52]. By assessing background pollutant exposure levels three months before and during each trimester of pregnancy and then by testing associations between corresponding exposure variables and lymphocyte immunophenotype distributions in cord blood, our study contributes to identify windows of exposure to air pollutants before and during pregnancy. So far only two studies have indicated that *in utero *exposure to urban air pollution is associated with immunodysregulation in newborns [20,21], and in these studies the levels of air pollution were higher than the levels encountered nowadays in most industrialized countries.

Our investigation is the first to consider the three-month period immediately preceding pregnancy, which involves the participation of epigenetic mechanisms in the development of the immune system. Recent epidemiological studies have provided evidence suggesting that environmental factors may cause epigenetic alterations affecting both the mother and her child [53]. Heritable epigenetic changes may disrupt the normal functioning of genes by affecting their expression without changes in the DNA sequence. Several epigenetic mechanisms have been suggested [54], including DNA methylation and histone modifications, which could determine or give a clear prediction of the future transcriptional state of the genome under the influence of environmental factors. Animal [55] and human [56,57] studies have indicated that *in utero *and early life environmental exposures may generate effects than can be inherited transgenerationally along with epigenetic modifications. An inverse association has been identified between prenatal lead exposure and genomic leukocyte DNA methylation (linked to transcriptional repression) in human cord blood [56]. Similarly, transplacental exposure to high levels of airborne PAHs has been related to aberrant DNA methylation changes, leading to dysregulation of gene expression and possibly childhood asthma [57]. Overall, these results suggest that maternal exposure to air pollutants during critical periods of prenatal development might lead to inappropriate gene expression and disease pathogenesis in later life by inducing epigenetic alterations in the fetus [58].

Several potential mechanisms may explain the links between *in utero *exposure and subsequent changes in cord blood cells. Several experimental studies have supported the hypothesis that antigenic cell stimulation by air pollutants and their metabolic by-products could be at the origin of the rise in some lymphocyte subsets by highlighting the disturbance of lymphocyte immunophenotype distributions by xenobiotics including tobacco smoke [59] and chemicals [60]. However, air pollutants could also act as adjuvants by amplifying specific responses towards other antigens such as viruses or allergens. Indeed it has been shown that diesel exhaust particles can amplify an allergen-specific Th2 response [61] but also enhance Th2 and Th1 cytokine production of primed T-cells recovered from asthmatics during exacerbation [62]. Such adjuvant effects could be involved in the increase of CD8+ and NK cells. Also, the depressed maternal immune system could allow for latent viral infections, potentially transmitted to the fetuses. This effect would indeed trigger an increase of NK cells and CD8+ T-cells, and hence a decreased CD4/CD8 ratio amplified by pollutants [38]. Lastly, animal experimental studies, investigating impacts of maternal exposure to diverse chemicals on the neonatal immune system [63], have indicated that the thymus, through its cellularity and functioning, may also play a role. Maternal exposure to certain chemicals during pregnancy has been associated with a reduction in thymic cellularity, thymic atrophy and an altered T-lymphocyte differentiation. During fetal immune system development, the thymus is the organ responsible for T-lymphocyte maturation. Lymphoid colonization starts from the tenth week of gestation and after twelve weeks, mature CD3+ T-lymphocytes are detectable in fetal blood. Therefore, a functional disturbance of the thymus may have serious consequences on lymphocyte immunophenotypes. Underneath these mechanisms there is the assumption that the fine and ultrafine particles can cross the alveolocapillary membrane into the systemic circulation [64] and also the placenta [65].

## Conclusions

Our data indicate that air pollution before and during pregnancy can be at the origin of disturbances in the functional balance between immune response mechanisms in the newborn. Although the biologic relevance of this finding is not entirely clear, the main observation is that for the first time at the general population level, exposure to major ambient air pollutants before and during pregnancy has been associated with a statistically significant alteration of the relative distribution of cord blood NK and T-lymphocyte phenotypes including a decrease in CD4+CD25+ regulatory T-cells. Whether alterations in developmental patterns of lymphocytes due to air pollution contribute to explain the rise in asthma and allergies - the so-called "allergy epidemics" observed in past decades - needs to be further investigated. Follow-up of the children in the EDEN cohort might shed light on this issue and is currently under way.

## Abbreviations

VOCs: volatile organic compounds; BETX: benzene: ethylbenzene: toluene: xylenes; CI: confidence interval; SD: standard deviation; NK: natural killer cells; CD19+: B-lymphocytes; BMI: body mass index; bf: before.

## Competing interests

The authors declare that they have no competing interests.

## Authors' contributions

NB and IAM conceived and planned the study. NB performed statistical analyses and data interpretation and wrote the manuscript. RS provided the air pollution data. MCB and AM contributed to the immunophenotyping and provided data on the immunological material. MAC was responsible for the human resource organization and contributed to the design of questionnaires. MNKS participated in the cord blood analyses and immunophenotyping. OT participated in the following up of the women during pregnancy and after delivery. GF supervised the group performing immunophenotyping. IAM supervised the project and assisted with writing the manuscript. All authors discussed the results and implications and commented on the manuscript. All authors read and approved the final manuscript.

## Pre-publication history

The pre-publication history for this paper can be accessed here:

http://www.biomedcentral.com/1471-2393/11/87/prepub
